# IL-17 signalling restructures the nasal microbiome and drives dynamic changes following *Streptococcus pneumoniae* colonization

**DOI:** 10.1186/s12864-017-4215-3

**Published:** 2017-10-23

**Authors:** Neil D. Ritchie, Umer Z. Ijaz, Tom J. Evans

**Affiliations:** 1 Institute of Infection, Immunity and Inflammation, University of Glasgow, Glasgow, UK; 2 School of Engineering, University of Glasgow, Glasgow, UK

**Keywords:** Innate immunity, inflammation, microbiome, nasopharynx, *Streptococcus pneumoniae*

## Abstract

**Background:**

The bacterial pathogen *Streptococcus pneumoniae* colonizes the nasopharynx prior to causing disease, necessitating successful competition with the resident microflora. Cytokines of the IL-17 family are important in host defence against this pathogen but their effect on the nasopharyngeal microbiome is unknown. Here we analyse the influence of IL-17 on the composition and interactions of the nasopharyngeal microbiome before and after pneumococcal colonization.

**Results:**

Using a murine model and 16S rRNA profiling, we found that a lack of IL-17 signalling led to profound alterations in the nasal but not lung microbiome characterized by decreased diversity and richness, increases in Proteobacteria and reduction in Bacteroidetes, Actinobacteria and Acidobacteria. Following experimental pneumococcal nasal inoculation, animals lacking IL-17 family signalling showed increased pneumococcal colonization, though both wild type and knockout animals showed as significant disruption of nasal microbiome composition, with increases in the proportion of Proteobacteria, even in animals that did not have persistent colonization. Sparse correlation analysis of the composition of the microbiome at various time points after infection showed strong positive interactions within the Firmicutes and Proteobacteria, but strong antagonism between members of these two phyla.

**Conclusions:**

These results show the powerful influence of IL-17 signalling on the composition of the nasal microbiome before and after pneumococcal colonization, and apparent lack of interspecific competition between pneumococci and other Firmicutes. IL-17 driven changes in nasal microbiome composition may thus be an important factor in successful resistance to pneumococcal colonization and potentially could be manipulated to augment host defence against this pathogen.

**Electronic supplementary material:**

The online version of this article (10.1186/s12864-017-4215-3) contains supplementary material, which is available to authorized users.

## Background

The microbial environment of the nasopharynx is diverse and medically important, as this is the usual niche of a number of important human pathogens such as *Neisseria meningitidis, Haemophilus influenzae* type B, and *Streptococcus pneumoniae* [1–4]. Nasal colonisation with such organisms precedes the development of disease and so understanding the dynamics of nasal colonisation and developing tools to control or limit nasal colonisation are important research goals. Host factors that affect colonization may alter immune defences against a pathogen but may also alter the composition of the resident microbiome [5, 6]. To be able to colonize initially, a pathogen not only requires the ability to overcome host defences but also to be able to compete effectively with the resident microbiota. Hence, it is important to understand the effects of host immune responses not only on the pathogen directly but also on the richness and diversity of the microbiome at the site of colonization, since this will influence the resistance and resilience to pathogen invasion [7].

The human pathogen *S. pneumoniae* (the pneumococcus) is an important worldwide cause of pneumonia, meningitis, sepsis, middle ear infections, and other invasive diseases, responsible for over 1 million deaths annually [8]. Colonization of the nasopharynx by this microbe is transient, usually less than 6 months, and will induce a specific antibody response directed at the carbohydrate capsule of the bacterium that is thought to contribute to clearance [2, 9]. Spread of the pathogen from the site of colonization in the nasopharynx to the lower airways is thought to proceed by micro-aspiration [10, 11]. In this site as well, the resident microbiome will also influence the ability of the pneumococcus to cause disease, and as with the nasopharynx, the composition of the lung microbiome will also potentially be altered by specific immune mechanisms.

Host and pathogen factors are important in pneumococcal colonization. Specific opsonophagocytic antibodies to the bacterial carbohydrate capsule are very effective at reducing colonization and induction of such antibodies by vaccination has been highly successful in reducing colonization by specific pneumococcal capsular serotypes [12]. However, cell-mediated immunity appears to be more important in naturally acquired immunity to pneumococcal colonization. In particular, CD4^+^ cells producing cytokines of the interleukin-17 (IL-17) family, Th17 cells, have been shown to be critical for primary clearance of colonizing pneumococci and subsequent secondary immune protection [13–15]. Several putative pneumococcal vaccines have been dependent on IL-17 mediated immunity for efficacy in mouse models [13, 16–18]. Although the immune mechanisms by which IL-17 provides defence against pneumococcal colonization have been well described, no studies have been made of the effects of this cytokine on the nasopharyngeal and lung microbiome before and after pneumococcal colonization. Changes in these bacterial populations will also influence whether an invading pneumococcal strain can compete with the resident microflora and become an established colonizer, and how rapidly it will be cleared. Competition between the invading pneumococcus, other competing pneumococcal strains and resident bacteria is also influenced by pathogen factors. Between-strain competition has been demonstrated in both animal models and human colonization [19–21]. For example, studies analysing the prevalence of double colonization with two different strains of pneumococci show that colonization with one strain is very inhibitory to acquisition of a second strain [21]. Bacteriocins produced by pneumococci have been shown to be important in mediating intraspecies competition in animal models [19]. Pneumococcal bacteriocins can also kill related bacterial species such as *Micrococcus* and *Lactococcus* species [22]; the biological importance of such activity in establishing colonization is not clear.

We hypothesised that IL-17 would be important in determining the composition of the normal nasopharyngeal and lung microbiome, and that following pneumococcal colonization, IL-17 would drive changes in nasopharyngeal microbiome composition. Additionally, we postulated that colonization by a pneumococcal strain would result in a reduced frequency of closely related bacteria within the Firmicutes phylum. Here, we use a murine model of experimental pneumococcal nasopharyngeal colonization to explore the influence of IL-17 on acquisition and carriage of this microbe, using mice with a targeted deletion of the common IL-17 receptor gene, *Il17ra*. Using 16S ribosomal DNA sequencing, we show that lack of IL-17 signalling produces profound effects on the nasopharyngeal but not the lung microbiome, with a loss in richness and diversity, expansion of Proteobacteria, and decreased abundance of Bacteroidetes and Acidobacteria. Following experimental inoculation of *S. pneumonia*e, in both wild type and *Il17ra* knockout (KO) animals there were multiple changes in the composition of the nasal microbiome, even in animals that did not show established colonization. In the lung, only the *Il17ra* KO animals showed changes in microbiome composition, with invasion of the inoculated pneumococcus but without evidence of clinical disease. Rather than inhibiting related Firmicutes, pneumococcal colonization promoted their presence while antagonising Proteobacteria. Given the risk of invasive disease resulting from colonization, these results reveal important host and pathogen determined changes in the nasal microbiome that could be exploited to limit pneumococcal disease.

## Results

### Experimental Set Up

To determine the contributions of IL-17 signalling to the composition and behaviour of the nasopharyngeal and lung microbiome before and after pneumococcal colonization, we utilized IL-17RA knockout mice and wild type mice, both bred in house and cohoused for 6 weeks after weaning as described in the Methods. Groups of mice were left untreated or inoculated with the SRL1 strain of pneumococcus in the anterior nares and the composition of recovered bacteria before and at various times after inoculation was analysed by 16S rDNA sequencing. Only 1 out of 96 total samples failed to amplify the 16S V1-V2 region but other samples were successfully amplified and sequenced with a median of 6.7 x 10^4^ (range 2.1-13.3) reads per sample. These were clustered into 2632 operational taxonomic units (OTUs). Rarefaction curves of the sample showed excellent depth, with all samples approaching an asymptote (Additional file 1: Figure S1A). Numerical estimate of sample coverage using the abundance-based coverage estimator [23] showed a median OTU coverage of 92.7%, with no significant difference between wild type and IL-17RA knockout animals (Additional file 1: Figure S1B).

To confirm that a lack of the IL-17RA receptor resulted in expected changes in IL-17 cytokine targets, we assayed for known IL-17 targets following SRL1 infection in wild type and IL-17RA knockout mice (Additional file 1: Figure S2). We confirmed that the known IL-17 target genes beta-defensin 4, S100A8, and macrophage inhibitory protein-1 alpha [24] were strongly induced following SRL1 infection in wild type animals, but their expression was significantly and strongly abrogated in IL-17RA knockout mice.

### Resting nasal microbiome

**Fig. 1.**
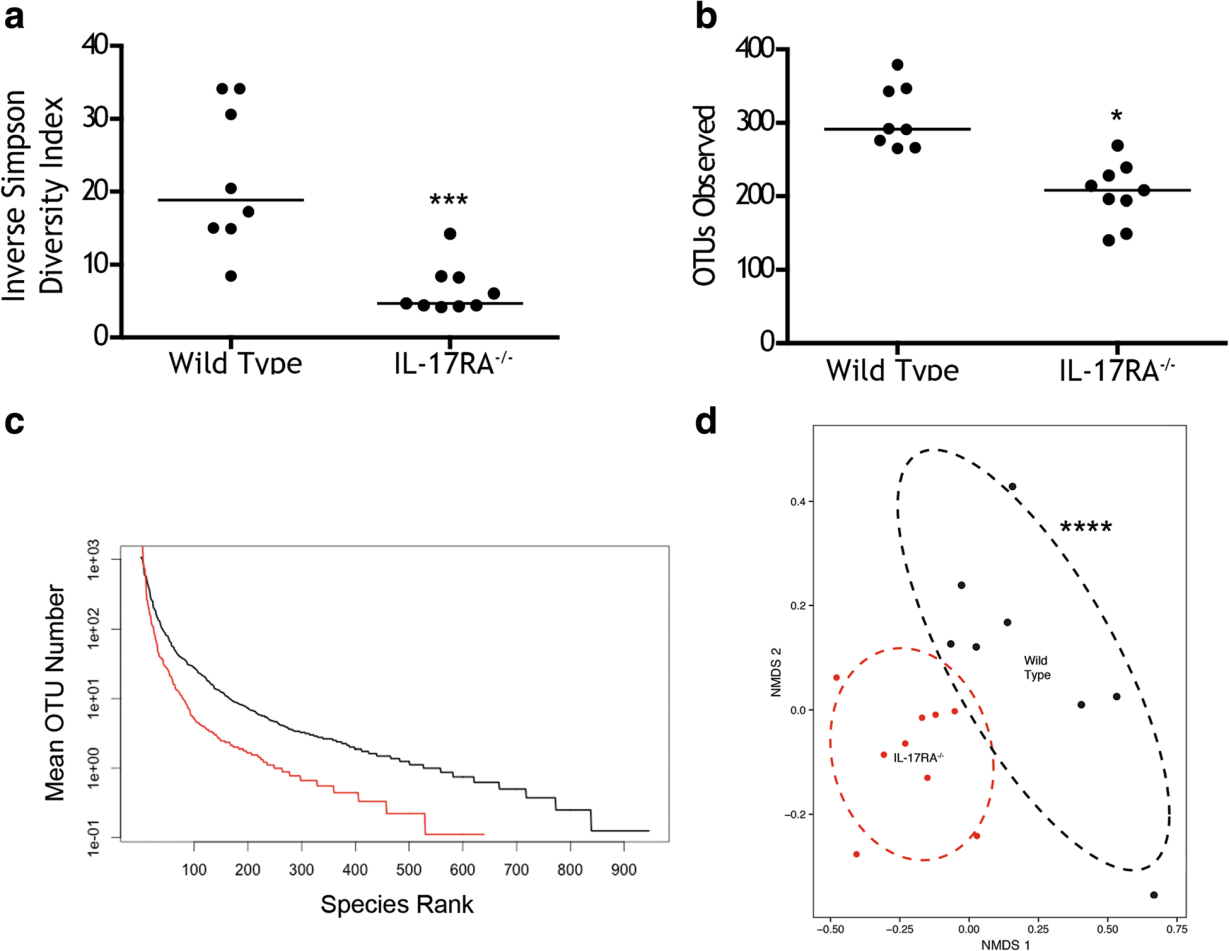
Analysis of resting microbiome of wild type and *Il17ra* KO mice. **a** Inverse Simpson Diversity Index and (**b**) number of observed OTUs between wild type and *Il17ra* KO mice (IL-17^-/-^). Each symbol is the result from an individual animal. Horizontal line denotes median. Significant differences between the groups are shown: ***, *p* < 0.001, * *p* < 0.05, Mann-Whitney test. **c** Rank abundance curves for the observed OTUs of WT (black) and *Il17Ra* knockout mice (red). **d** Non-metric multidimensional analysis with ellipses representing 80% confidence limit for each group. Differences between the groups were significant (***. *P* < 0.0001, using PERMANOVA)

The wild type nasal microbiome had a median of 292 OTUs observed per sample. The microbiome was principally composed of Proteobacteria (35.7%) and Firmicutes (30.0%) with smaller populations of Bacteroidetes (13.2%) and Actinobacteria (10.6%). A number of recognised mouse pathogens were present including *Streptococcus*, *Staphylococcus* and *Pasteurella sp.,* and these were present in all mice studied. α-diversity among *Il17ra* KO mice was significantly lower compared to wild type as measured using the inverse Simpson diversity index (Fig. 1a). OTU count (richness) was also significantly lower in *Il17ra* KO mice (Fig. 1b). OTU abundance curves of the wild type and IL-17RA KO animals show the clear differences in richness and evenness between the two groups (Fig. 1c). Ordination using two-dimensional non-metric multidimensional scaling analysis (NMDS) based on the Bray-Curtis dissimilarity index between each animal sample, showed clear separation of the two groups that was highly statistically significant (Fig. 1d), using permutational multivariate analysis of variance (PERMANOVA) [25] .

**Fig. 2.**
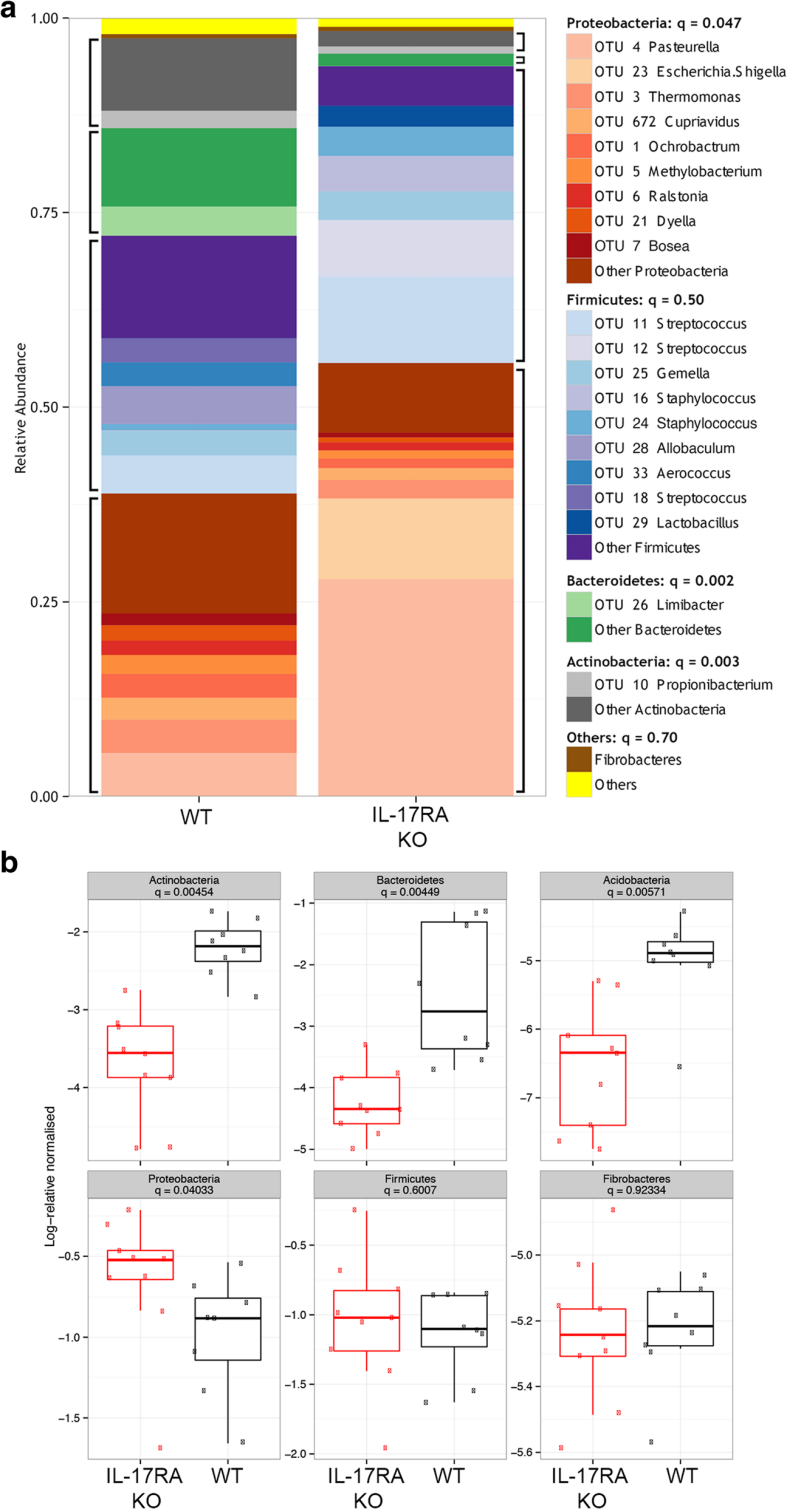
Analysis of resting wild type and *Il17ra* KO microbiome. **a** The 20 most abundant OTUs from the resting dataset are plotted individually with the remainder combined. Data are pooled from several mice (n = 8-9/group) and blocks of similar colours represent common bacterial phyla. Significance testing using Kruksal-Wallis test corrected for multiple comparisons is shown in the legend. q value represents outcome of testing for difference in abundance of major phyla between wild type and *Il17ra* KO mice. **b** Plots showing log normalised relative abundance of the 6 most abundant bacterial phyla. Points are individual animals. Box encloses the interquartile range, line is the median and the whiskers extend to 1.5 x above and below the interquartile range

Distribution of bacterial phyla and individual OTUs between the wild type and IL-17RA knockout animals is summarized in Fig. 2. Individual OTUs within phyla are shown in Fig. 2a. Compared to wild type, *Il17ra* KO mice had increased abundance of Proteobacteria and decreased abundance of Bacteroidetes, Actinobacteria, and Acidobacteria (Fig. 2b). Notably, however, there was not a difference in the relative abundance of Firmicutes, the phylum containing Streptococci, between the two groups of animals. The decreased diversity of *Il17ra* KO mice was reflected in small numbers of OTUs dominating the nasal microbiome of these animals. The three most abundant OTUs (OTT 4: Pasteurella, OTU 11: Streptococcus and OTU 23: Escherichia/Shigella) had a combined abundance of 49.4% whereas the 17 most abundant OTUs would be required to reach the same combined abundance in wild type mice. OTU23 was not one of the 20 most abundant OTUs in the wild type mice but as shown in Fig. 2a, was a significant component of the nasal microbiome in the IL17RA knockout animals.

### Resting Lung Microbiome

**Fig. 3.**
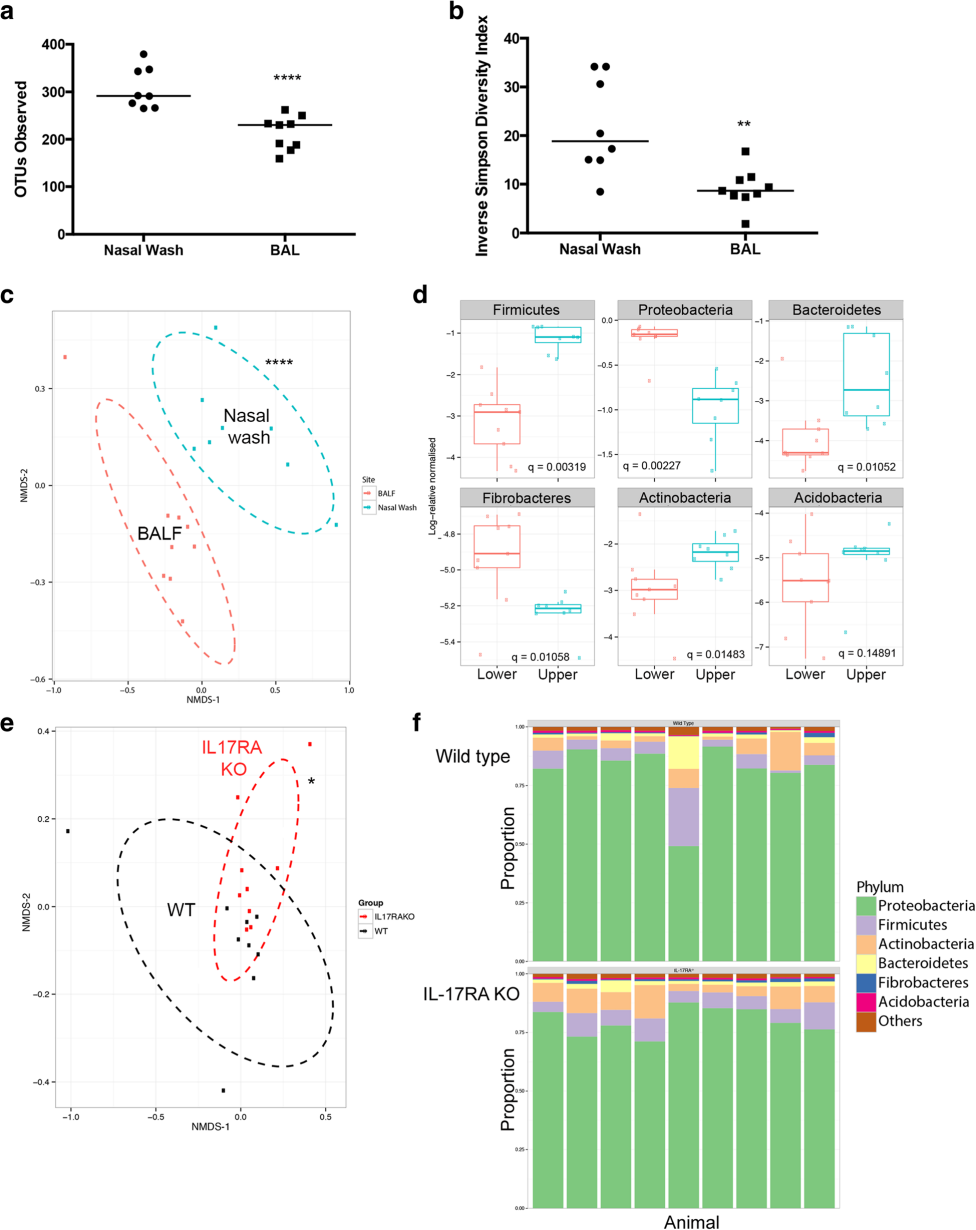
Analysis of lung microbiome. **a** OTU count and (**b**) Inverse Simpson index in samples rarefied to even sequencing depth. **c** Multidimensional scaling analysis of samples as indicated with ellipses indicating 80% confidence of sample distribution. **d** Plots showing log normalised relative abundance of the 6 most abundant bacterial phyla. **e** Multidimensional scaling analysis of lung microbiomes from groups as indicated, with ellipses indicating 80% confidence of sample distribution. **f** Composition of lung microbiome grouped by phylum for individual animals. All symbols, statistical tests and significance levels as in Fig. 1

There was a marked difference between nasal and the lung microbiome, as shown in Fig. 3. Both richness and diversity was significantly less in the lungs than in in the nasal microbiome (Fig. 3a and b). NMDS analysis showed the microbiome composition between the two sites is considerably different (Fig. 3c). In particular, Proteobacteria (the most abundant phylum in the lung microbiome) were considerably more abundant in the lung compared to the nasal site. Fibrobacteres were also significantly more abundant; Firmicutes, Bacteroidetes and Actinobacteria were all significantly less abundant in the lung compared to the nasal site (Fig. 3d).

Next, we compared the composition of the lung microbiome in wild type mice compared to the IL-17RA knockout animals (Fig. 3e). In contrast to the nasal samples, there was considerable overlap in the composition of the samples from the two groups, although the differences observed were significant. At phylum and family level, no significant differences between the two groups of animals were found (shown for phyla in Fig. 3f).

### Nasal colonisation with S. pneumoniae

**Fig. 4.**
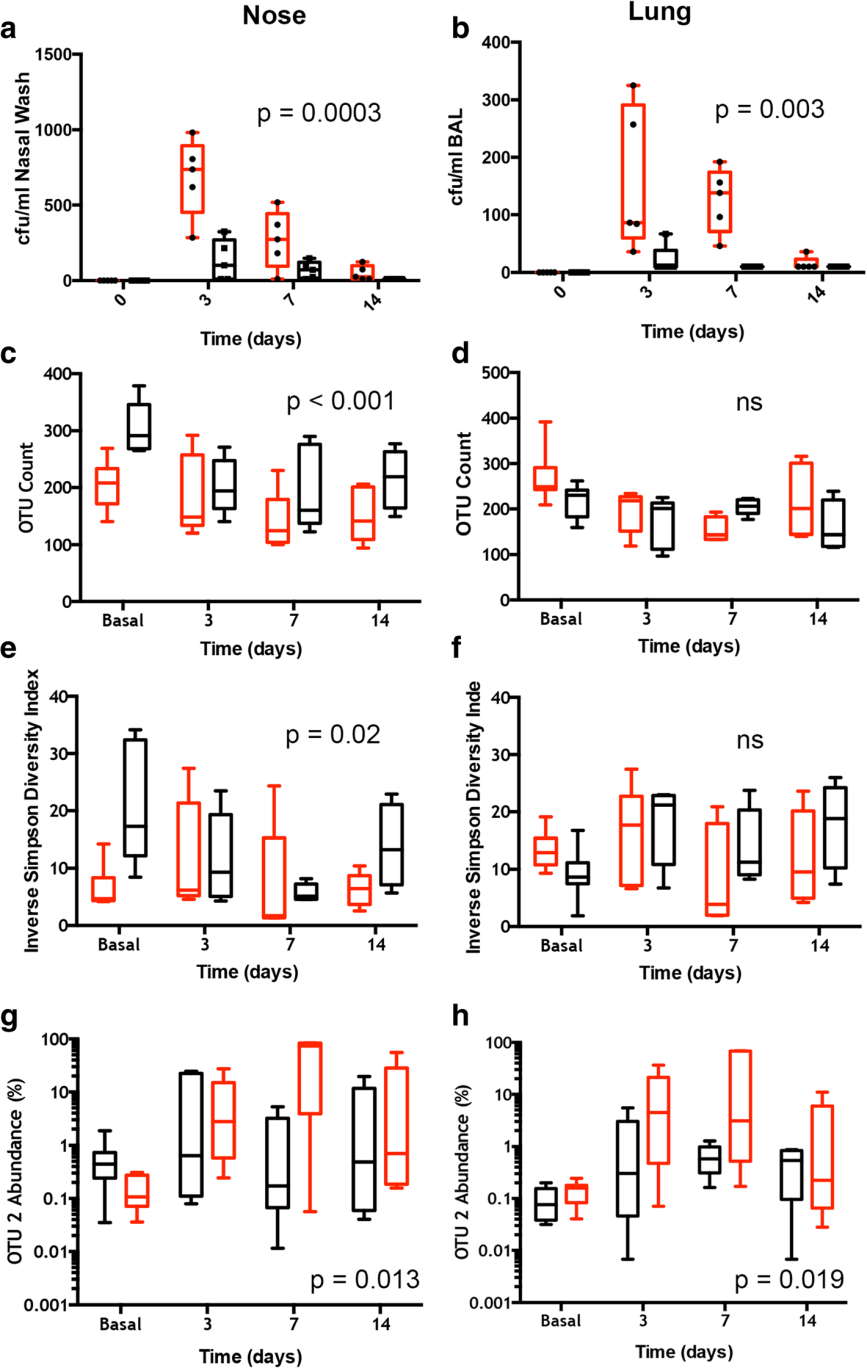
Comparison of nose and lung microbiome following Pneumococcal Inoculation. **a** Nasal and (**b**) lung colonisation with *Streptococcus pneumoniae* assessed by routine culture methods. Differences between the groups were significant, 2-way ANOVA, (**a**, p=0.0003, **b**, p=0.003). Nose (**c**, **e** and **g**) and lung (**d**, **f** and **i**) microbiomes compared for OTU count (**c** and **d**), Diversity (**e** and **f**) and OTU2 abundance (**g** and **h**). Symbols for box and whisker plots as in Fig. 1. *P* values are for 2-way ANOVA between the wild type and *Il17ra* KO animal groups. ns = not significant

Mice were inoculated with the SRL1 strain of pneumococcus and the nasal and lung microbiome analysed by 16S ribosomal DNA analysis at days 3, 7 and 14. Conventional culture analysis of nasal wash of mice colonised with *S. pneumoniae* are shown in Fig. 4a. No mice had any colonies of *S. pneumoniae* on nasal wash prior to experimental exposure. Inoculation with 5 x 10^5^ cfu/mouse induced low level colonisation in both wild type and *Il17ra* KO mice which peaked at 3 days following inoculation, and then fell over the 14 days of the experiment. *Il17ra* KO mice had significantly higher colonisation density in the nose than wild type animals (P < 0.001, 2-way ANOVA). Similarly, lung colonization by SRL1 peaked at 3 days after inoculation and fell thereafter (Fig. 4b). The difference between wild type and IL-17RA knockout mice was significant (p < 0.003, 2-way ANOVA). In a separate experiment, we monitored the neutrophils within the nasal wash before and after introduction of pneumococci. Prior to inoculation, both groups of animals showed a low percentage of recovered neutrophils (Additional file 1: Figure S3). 7 days following introduction of pneumococci, the percentage of neutrophils increased in both sets of animals, but neutrophils in the nasal wash were significantly lower in the *Il17ra* KO animals compared to the wild type controls (Additional file 1: Figure S3).

### Nasal and Lung microbiome following colonisation

OTU count (richness) following nasal colonisation is shown in Fig. 4c for the nasal microbiome and Fig. 4d for the lung microbiome. In the nasal microbiome, wild type animals showed a drop in OTU count following pneumococcal inoculation that partially recovered by day 14. Conversely, the IL-17RA knockout animals showed a lower initial OTU count that dropped to a small degree following pneumococcal inoculation (Difference between the groups significant, p< 0.001, 2-way ANOVA). In the lung, there was a slight reduction in diversity following inoculation; there was no significant difference between the wild type and IL-17RA knockout animals (Fig. 4d). Wild type animals experienced a fall in inverse Simpson diversity index (Fig. 4e)) following nasal colonisation which appeared to be reversing by day 14. In contrast, there was little difference in inverse Simpson diversity index among *Il17ra* KO mice throughout, a significant difference between the groups (*p* < 0.001, 2-way ANOVA). In the lung, there was little change in diversity through the experiment and no significant difference between the animal groups (Fig. 4f).

OTU2 was identified as having an identical sequence to the SRL1 pneumococcal strain. Low (< 1%) levels of OTU2 were found in nasal wash samples from mice that had not been inoculated with SRL1. Pneumococcus is not a normal pathogen of mice, and no colonies of pneumococcus were ever recovered from nasal wash of these animals. Moreover, using amplification of the capsular polysaccharide gene *cpsA*, a highly specific marker for *S. pneumoniae*, we never found any signal in nasal washes from unexposed mice, but consistently found that samples from inoculated mice were positive by this assay (Additional file 1: Figure S4). The closest ribosomal sequence to OTU2 in our data set were from species of viridans streptococci which we conclude are responsible for the low-level signal from OTU2 seen in control uninoculated animals. Thus, we conclude that the OTU2 signal in control uninoculated mice is from viridans group streptococci and that the marked expansion of OTU2 following SRL1 inoculation indicates the presence of this added organism.

The abundance of OTU2 following inoculation with SRL1 is shown in Fig. 4g and h in nose and lung respectively. The abundance in both sites increased markedly following inoculation, peaking at 7 days after inoculation. In both sites, levels of this OTU were significantly higher in the IL-17RA knockout animals compared to wild type (2-way ANOVA, p < 0.05 for both sites).

**Fig. 5.**
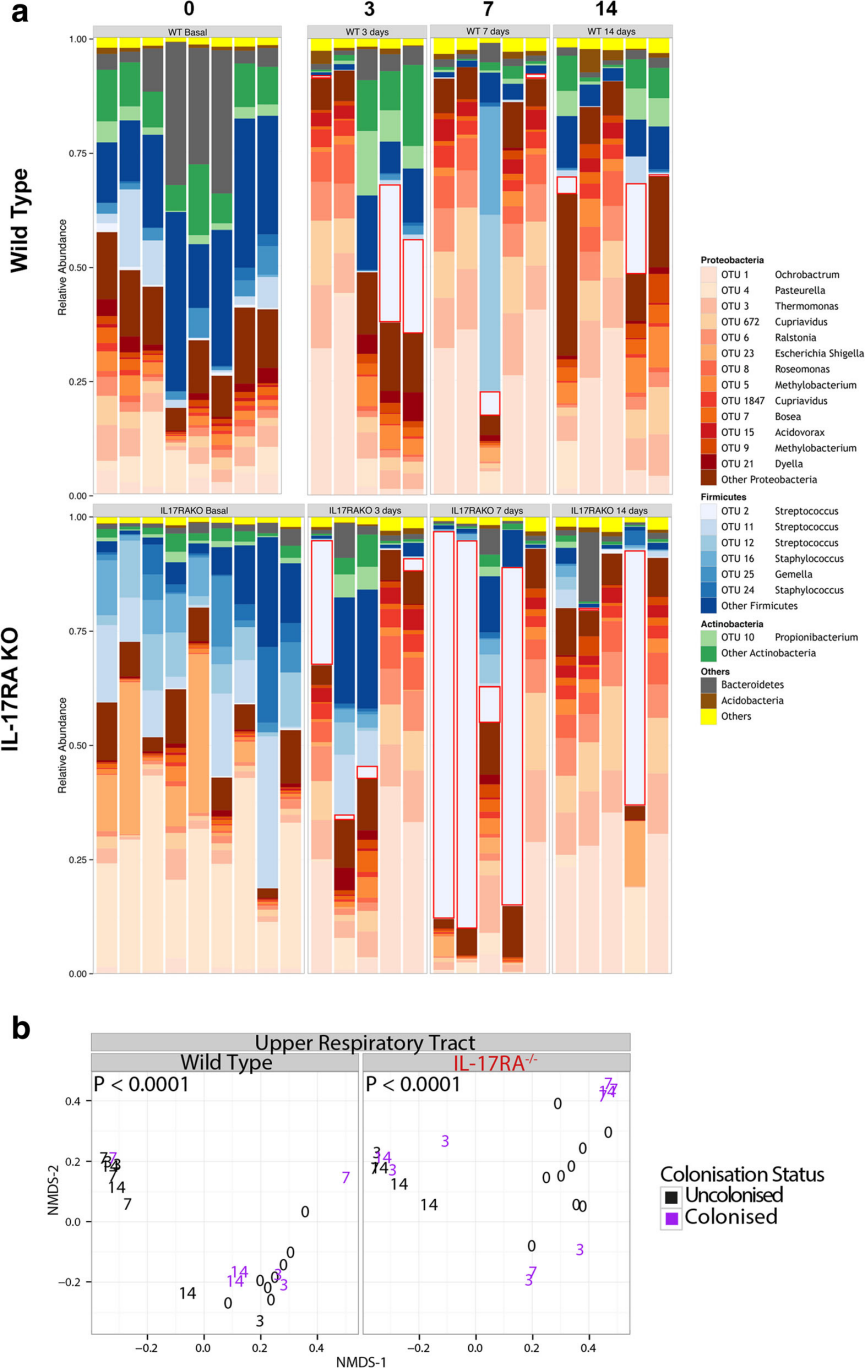
OTU level analysis of individual nasal microbiomes of wild type and *Il17ra* KO mice with and without artificial inoculation with *S. pneumoniae*. **a** The 20 most abundant OTUs from the dataset are plotted individually with the remainder combined. Data are grouped as unchallenged (0) or days following inoculation. Blocks of similar colours represent common bacterial phyla. OTU 2 is the cluster containing the expected sequence of the strain (SRL1) used in these experiments and is outlined in red. **b** 2 dimensional NMDS plots of the nasal microbiome before and after pneumococcal inoculation. Each animal is plotted as a number corresponding to the time (days) when the sample was taken after pneumococcal inoculation. *P* values are for PERMANOVA testing of differences between animals before and after pneumococcal introduction

Changes in the relative abundance of the top 20 most abundant OTUs within the nose following pneumococcal colonization are shown in Fig. 5a. This shows the heterogeneity of responses in individual animals. However, some clear patterns are evident. Most wild type mice had low abundance (<10%) of OTU 2 and no mouse exceeding 25%. In contrast, several *Il17ra* KO mice had high abundances of OTU 2 exceeding 50%; these results are summarized in Fig. 4g. Even in animals that did not have high levels of OTU2 colonization, there were marked changes in their microbiome composition. For both wild type and *Il17ra* KO mice, there was a significant difference in the microbiome composition following pneumococcal inoculation, shown using non-metric multidimensional scaling (Fig. 5b). In both wild type and in *Il17RA* knockout animals, there is an expansion in proteobacteria in animals that do not show significant colonization with OTU2 (Additional file 1: Figure S5A). This was also true in the IL-17RA knockout animals (Additional file 1: Figure S5A), showing that this response is not driven by IL-17.

**Fig. 6.**
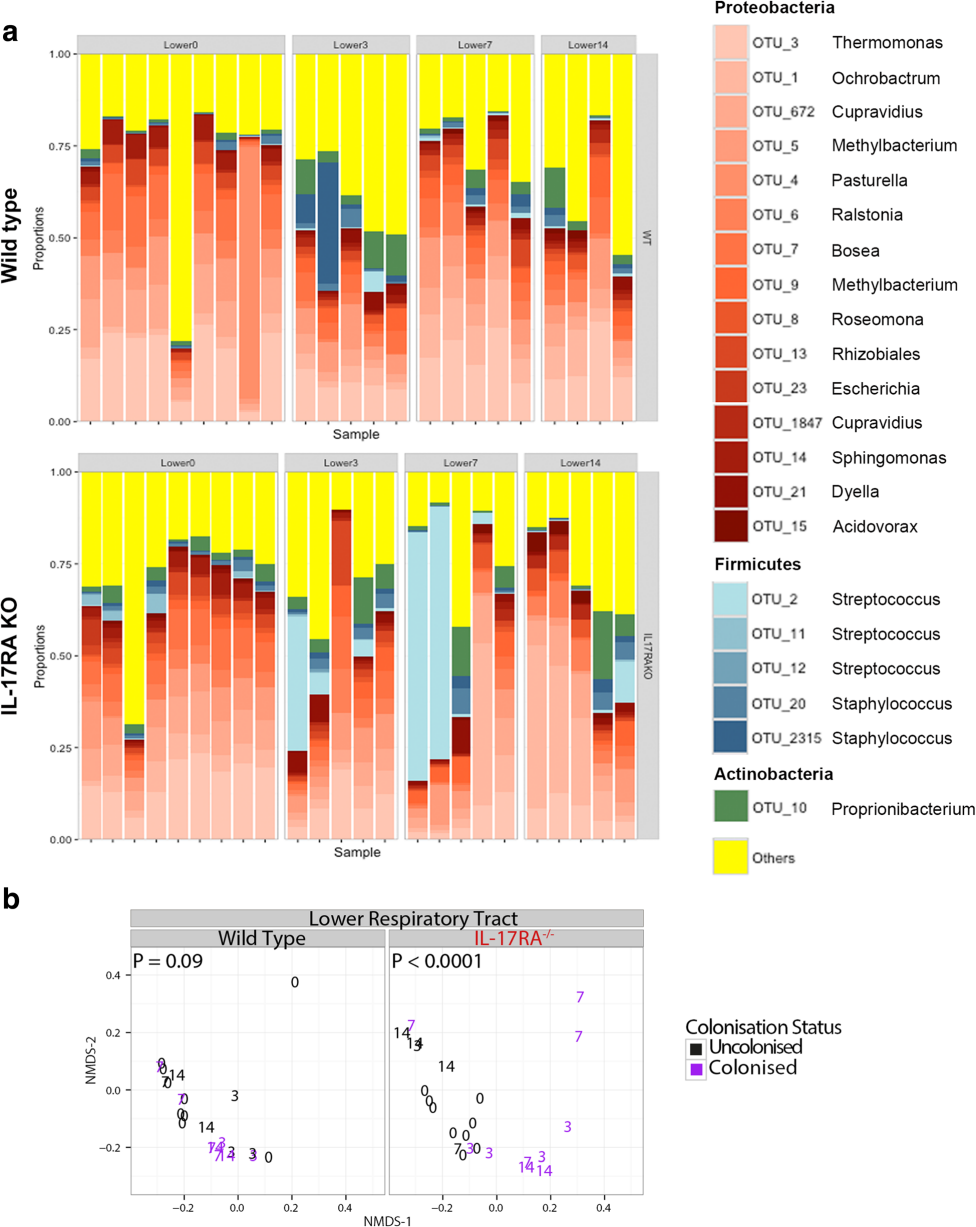
OTU level analysis of individual lung microbiomes of wild type and *Il17ra* KO mice with and without artificial inoculation with *S. pneumoniae*. **a** Data plotting as in Fig. 5a but for the lung microbiome. **b** as Fig. 5b but for the lung microbiome

Changes in the relative abundance of the top 20 most abundant OTUs within the lungs following pneumococcal colonization are shown in Fig. 6. In wild type animals, there was no significant difference in microbiome composition following pneumococcal inoculation (Fig. 6a and b). However, there was a significant shift in the composition of the lung microbiome of the *Il17ra* KO mice following the introduction of pneumococci (Fig. 6a and b). Wild type animals showed little evidence of OTU2 introduction into the lung microbiome. However, in the *Il17ra* KO mice, some of the colonized animals showed a marked preponderance of OTU2, particularly at day 7 after inoculation (as summarized in Fig. 4h). These levels correlated with the presence of OTU2 in the nose of the same animal at the same time. Despite this preponderance of OTU2 within the lung microbiome, these animals remained completely well with no signs of any disease.

### Sparse Correlation Analysis of Nasal Microbiome Before and After Pneumococcal Colonization

**Fig. 7.**
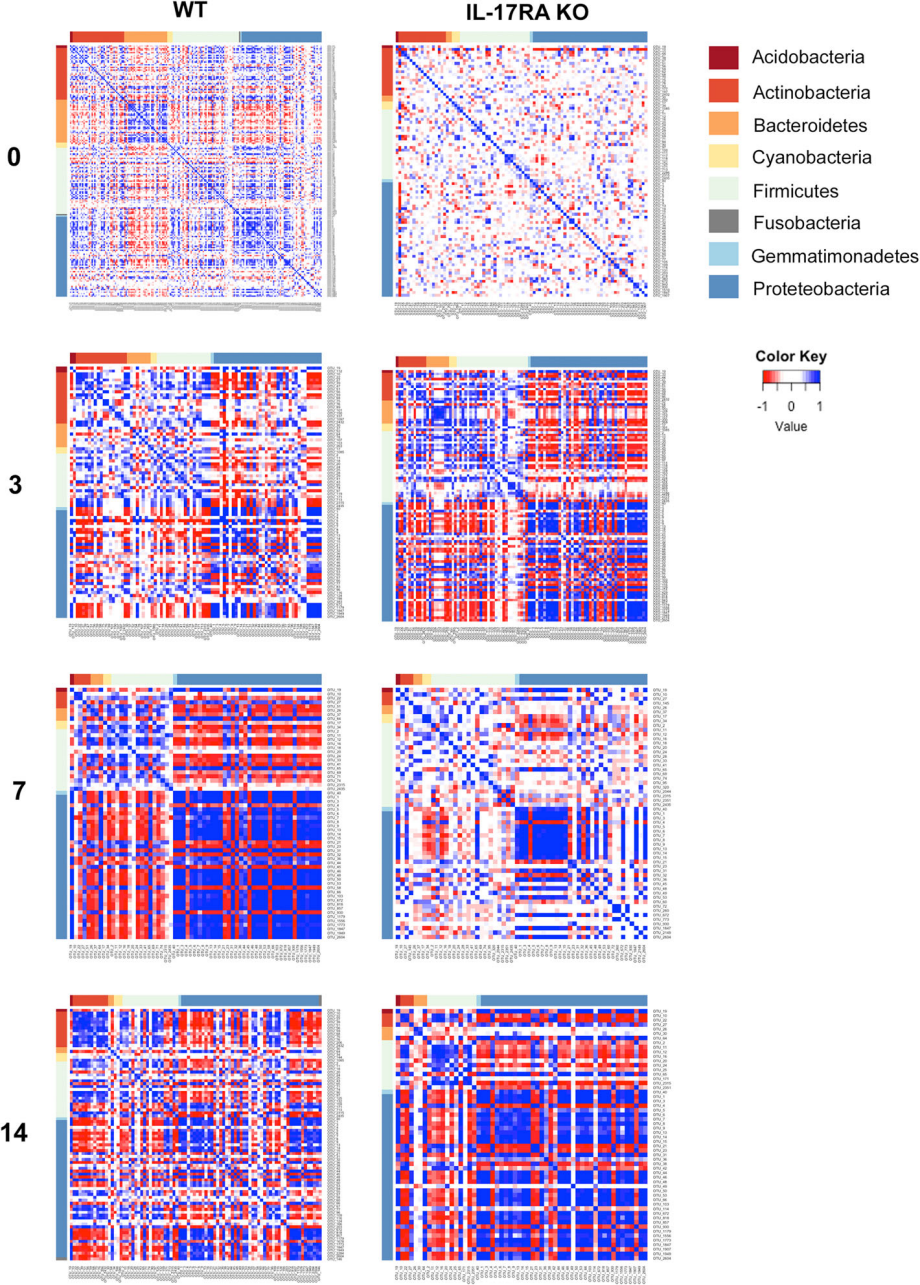
Sparse correlation analysis of nasal microbiome before and after pneumococcal colonization. OTUs with more than 500 reads from the normalised data were analysed by SparCC at time 0 and at the indicated times after pneumococcal inoculation. Each panel shows SparCC correlation coefficients between each OTU colour coded as indicated between -1 and 1. OTUs are grouped by phylum as shown

In order better to understand the multiple interactions between the different bacterial populations within the nasopharynx, we performed a sparse correlation analysis of the different bacterial OTUs before and after colonization, using the SparCC network inference tool that corrects for the compositional nature of microbiome data (Fig. 7). This approach is important since conventional correlations between organisms in compositional microbiome analysis is prone to error, since expansion of one OTU will perforce reduce the percentage of the other OTUs even if there is no direct interaction with these other microbes.

In both wild type and IL-17RA knockout animals, there is little evidence of any significant correlation between OTUs in resting animals (Fig. 7). Following SRL1 inoculation, there was a noticeable change in the observed sparse correlations between different OTUs. In wild type animals, there is an increase in the proportion of Proteobacteria which show a marked positive correlation between the majority of OTUs within this phylum. This is most evident at 7 days after SRL1 inoculation and is returning to baseline at 14 days. Similarly, there is a positive correlation between most OTUs of the Firmicutes, again most evident at day 7. Correspondingly, there was a notable negative correlation between OTUs within the Firmicutes and those of the Proteobacteria, most marked at day 7 and returning to baseline at day 14. In the IL-17RA knockout animals, there was a similar pattern of positive correlations within the Proteobacteria noticeable at 3 and 7 days and even more marked at day 14. Positive correlations within the Firmicutes were also evident, again with different kinetics to the wild type animals, and still very evident at 14 days after SRL1 inoculation.

**Fig 8.**
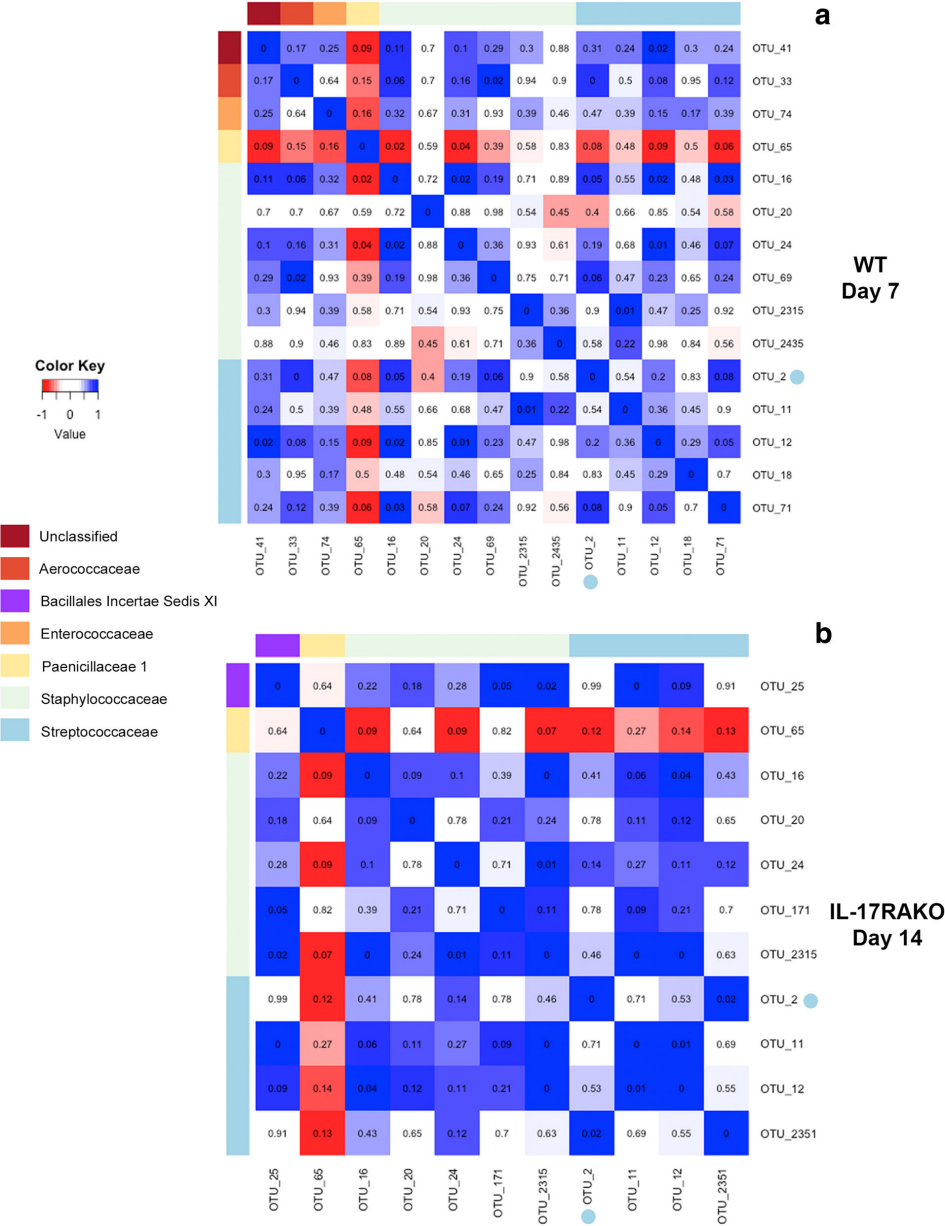
Sparse correlation analysis of Firmicutes within nasal microbiome after pneumococcal colonization at day 7 in WT animals (**a**) and IL-17RA knockout (KO) animals at day 15 (**b**). As Fig. 7, but just showing OTUs belonging to the Firmicutes and grouped by family as shown. OTU2, the infecting pneumococcal strain is shown highlighted with a blue dot. Inserted figures are pseudo-*p* values showing the proportion of 100 resampled matrices of the original data that had SparCC correlation values more extreme than shown here; *p* < 0.05 was taken as significant

We explored the positive correlations observed within the Firmicutes in more depth (Fig. 8), for wild type animals at day 7 (Fig. 8a) and for the IL-17RA knockout animals at day 14 (Fig. 8b); these were the times showing the maximal positive correlations within the phylum. The marked positive correlations between most of the different members of the Firmicutes are seen for both groups of animals. The figures within the boxes show the *p* values of the correlations as described in the Methods. Although few of these reach significance, (*p* < 0.05), only 1 OTU (OTU65) shows any sign of a negative correlation (< -0.5), the majority of other interactions between the members of the Firmicutes in both WT and IL-17RA knockout animals at the time points shown are positive (correlation > 0.5). This is a highly significant difference from a calculated expected value based on an even distribution of correlation values (*p* < 0.0001, Chi-squared on proportions for both WT at day 7 and IL-17KO at day 14). Thus, the presence of OTU2 generally acts to promote the presence of other bacterial OTUs within the Firmicutes. We also found a significant positive correlation between the density of SRL1 colonization and the relative proportions of other Firmicutes (Additional file 1: Figure S5B). Thus, these results suggest our hypothesis that colonization with *S. pneumoniae* would reduce the abundance of related Firmicutes through inter-specific competition through agents such as bacteriocins is not true. The significance of this is considered further in the Discussion.

**Fig. 9.**
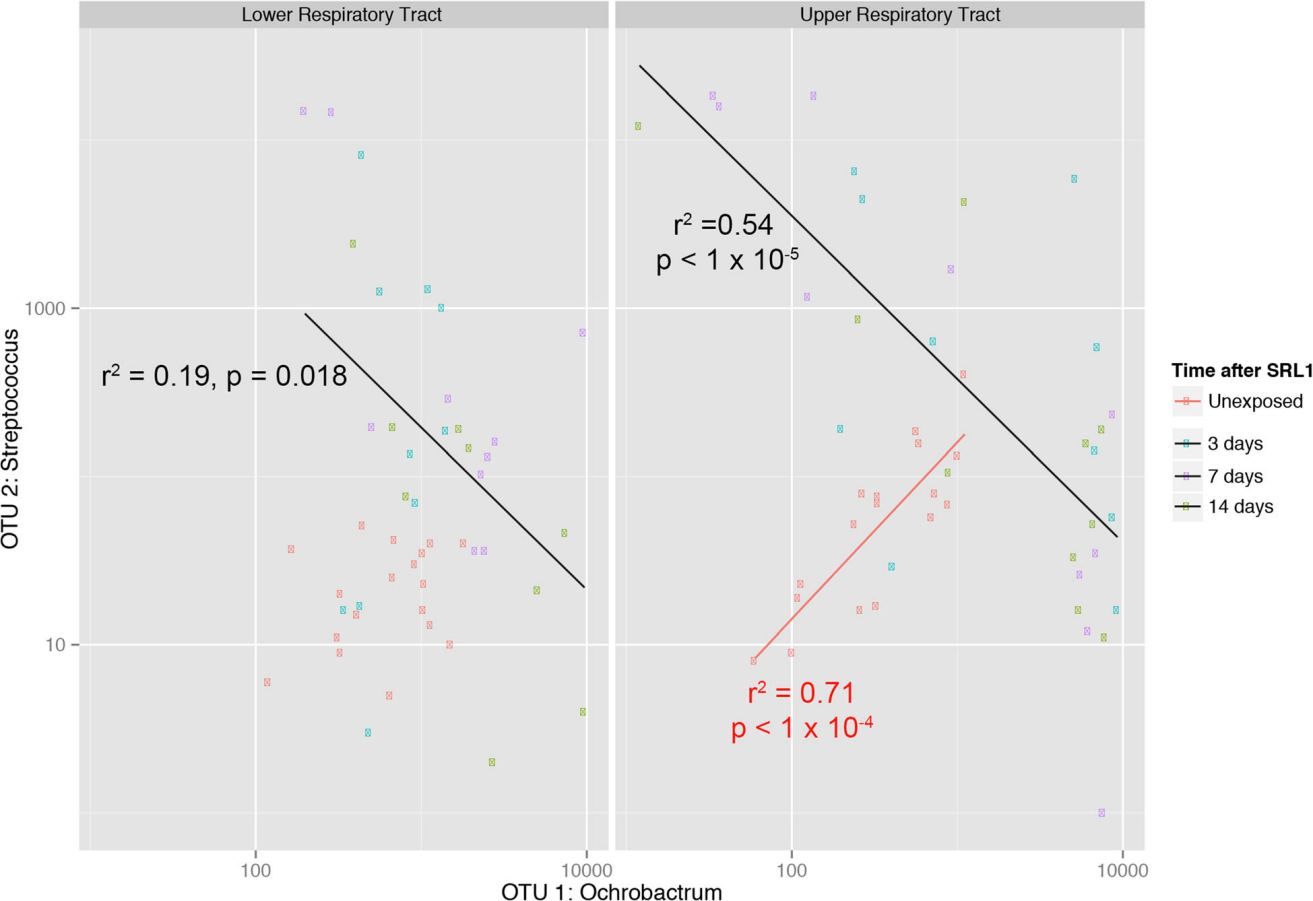
Association between OTU2 (Streptococcus including SRL1) and OTU1 (Ochrobactrum). Normalized abundance data for OTU 2 and OTU1 is plotted with generalised linear modelled lines of best fit shown for exposed (black) and non-exposed (red) mice. No line of best fit is shown for the unexposed, lower respiratory tract samples because there was no evidence of a correlation. R^2^ and *p* values for each line of best fit are as shown

In both the lung and the nose microbiome, we noted that after inoculation with SRL1, some animals showed an increase in the proportion of OTU1, an *Ochrobactrum* species. These were animals that in general had very low levels of OTU2. We tested this negative correlation between OTU1 and OTU2 by fitting a generalized linear model to the rarefied abundance data of OTU1 and OTU2 (Fig. 9). In the upper respiratory tract of un-exposed animals, there was a strong positive correlation between OTU1 and OTU2 (*P* < 0.0001) whereas among exposed animals the correlation was strongly negative (*P* < 0.0001). A similar pattern was repeated in the lower respiratory tract samples among exposed mice (*P* < 0.0001), although in this case there was no correlation among unexposed mice (*P*=0.25).

### *Differences in Gene Function between Wild Type and IL-17RA* KO *Nasal Microbiomes*

Finally, we compared the gene function of the nasal microbiome between wild type and IL-17RA knockout animals using Tax4Fun as described in the Methods to infer gene composition from the 16S amplicon analysis. KEGG Orthology Classifications between the two groups of animals are shown in Table 1. There were significant increases in the IL-17RA knockout animals in mapped genes belonging to the Glycan biosynthesis and metabolism and Infectious Diseases: Bacterial categories, with significant reductions in three other categories as shown. The Infectious Diseases: Bacterial category includes genes known to be important in *E. coli* infection. Together with the data showing the increased abundance of Proteobacteria in the nasal microbiome of the IL-17RA knockout mice (Fig. 2b) suggests IL-17 actions normally act to resist the presence of microbes such as *E. coli* in the nasal microbiome.

**Table 1:** Frequency of KEGG Orthologs in the imputed metagenome of the murine nasal microbiome calculated using Tax4Fun

KEGG Orthology Classification	Abundance of mapped genes, % (IQR)		q value
	Wild Type	IL-17RAKO	
Metabolism			
Carbohydrate metabolism	13.16 (0.6)	13.59 (0.16)	0.27
Energy Metabolism	7.22 (1.14)	6.35 (0.37)	0.24
Lipid metabolism	3.48 (0.36)	3.45 (0.04)	0.71
Nucleotide metabolism	6.27 (0.38)	6.54 (0.43)	0.27
Amino acid metabolism	11.25 (0.39)	10.78 (0.35)	0.27
Metabolism of other amino acids	2.69 (0.08)	2.73 (0.09)	0.27
Glycan biosynthesis and metabolism	**2.85 (0.13)**	**3.36 (0.26)**	**0.0016**
Metabolism of cofactors and vitamins	8.28 (2.01)	7.63 (0.22)	0.45
Metabolism of terpenoids and polyketides	**2.89 (0.15)**	**2.40 (0.17)**	**0.0074**
Metabolism of other secondary metabolites	**0.767 (0.08)**	**0.651 (0.09)**	**0.0016**
Xenobiotics degradation and metabolism	3.41 (0.78)	2.78 (0.43)	0.039
Genetic information processing			
Transcription	0.275 (0.04)	0.273 (0.04)	0.75
Translation	5.95 (0.76)	6.05 (0.97)	0.69
Folding, sorting and degradation	2.75 (0.33)	2.87 (0.27)	0.28
Replication and repair	5.55 (0.65)	5.71 (0.62)	0.56
Environmental information processing			
Membrane transport	11.09 (1.59)	12.51 (0.5)	0.13
Signal transduction	5.60 (1.17)	5.61 (1.09)	0.77
Signaling molecules and interaction	0.000276 (0)	0.000106 (0)	0.025
Cellular processes			
Transport and catabolism	0.266 (0.07)	0.259 (0.02)	0.68
Cell motility	1.27 (0.6)	1.03 (0.62)	0.28
Cell growth and death	**1.52 (0.08)**	**1.39 (0.04)**	**0.0016**
Human disease			
Infectious Diseases: Bacterial	**1.84 (0.14)**	**2.42 (0.29)**	**0.0019**

Differences were analysed using Mann-Whitney tests corrected for multiple comparisons using the method of Benjamini, Krieger and Yekutieli with a false discovery rate of 1%. Significant differences between Wild type and IL-17RAKO animals are shown in bold ( q value < 0.01)

## Discussion

Much more is known of the gastrointestinal microbiome compared to that of the nose. Studies have shown that the microbiome at this site resembles the skin microbiome [1] and is distinct from the lung microbiome, which would appear to be derived from the oral bacterial communities [26, 27]. Immediately following birth, the nasopharyngeal microbiome in humans is composed of a mixed community derived from many different niches [28]. Soon thereafter, however, the composition of this site differentiates into a more stable skin-like community. Mode of delivery has some limited impact on the dynamics of this process [28]. In our study, the resting nasopharyngeal microbiome was very similar between co-caged individuals of the same genotype but with significant differences between wild type and *Il17ra* KO mice. In contrast, human twin studies suggest that host genetics play little role in the composition of the nasal microbiota [29]. However, such studies cannot give information as to the effect of defined genetic differences in key immune response pathways as studied here. Indeed, another study investigating the role of type III interferon in susceptibility to *Staphylococcus aureus* superinfection found a significant impact of deletion of the gene encoding the type III interferon receptor on the composition and dynamics of the murine nasal microbiome following influenza infection [5]. In contrast to the differences observed in the nasal microbiome between wild type and *Il17ra* KO animals, we found very similar bacterial communities in the lungs of these animals.

Our experiments utilised animals with a genetic defect in IL-17RA, the common receptor subunit for IL-17 family cytokines. Previous work has shown such animals are defective in pathogen-induced IL-17 signalling [30] and other studies have shown the lack of IL-17 RA abolishes response to IL-17A [31]. We also demonstrate here that following SRL1 infection, known IL-17 cytokine targets are not upregulated in IL-17RA knockout animals compared to wild type controls (Additional file 1: Figure S2). Taken together, we thus feel that the changes in microbiome we observe in IL-17RA knockout mice directly reflect lack of IL-17 cytokine signalling.

Our study shows that lack of IL-17 signalling alters the resting nasal microbiome, leading to increased proportion of Proteobacteria, with reductions in Bacteroidetes, Actinobacteria and Acidobacteria, with a reduction in overall microbiome diversity and evenness. This suggests that IL-17 effectors such as neutrophils and antimicrobial peptides [32] have a significant role in maintaining the normal microbiome composition, in particular reducing colonization with potentially pathogenic Proteobacteria, such as *Pasturella* and *Escherichia* species. The differences observed in the *Il17ra* KO animals suggest also that there may be a low level of inflammation at this site in response to exposure to environmental bacteria or particulate matter, which is in part mediated by IL-17. The resting lung microbiome was distinct from the nasal microbiome and did not differ appreciably between wild type and *Il17ra* KO animals. This may reflect the lack of inflammation in resting animals, where the lung is protected from environmental insult by the mucociliary action of the respiratory mucosa.

Following inoculation of the nasopharynx with pneumococci, there were marked changes in both the nasal and lung microbiome both within and between the wild type and *Il17ra* KO animals. This was noticeable not only in those animals that showed ongoing presence of the pneumococcus within the nose but also in those animals that had cleared the bacterium. Notably, there was an expansion of Proteobacteria at the expense of the Firmicutes in mice that had cleared the nasal pneumococcal inoculum, both in wild type and *Il17ra* KO animals. This may reflect the inflammatory response induced by the introduction of pneumococci, as has been noted in many studies of the effect of inflammation in the composition of the gut microbiome [33, 34]. A dominant mechanism would appear to be the induction of inducible nitric oxide synthase in the mucosa, leading to increased nitrite and nitrate in the luminal fluid. In the gut, these anions can be used as terminal electron acceptors for the respiratory chain in many Proteobacteria, allowing them to proliferate at the expense of obligate anaerobic Firmicutes which cannot use these nitrogen containing anions as electron acceptors [35, 36]. Although it might seem that the aerated nasal passages would be a predominantly aerobic environment, in our study we recovered significant proportions of strictly anaerobic Bacteroidetes in the resting nasal microbiome, as has been noted on other studies [26, 27]. The niche for such anaerobes may be within nasal mucus, nasal glands or at the base of nasal hairs. Thus, we propose that the bloom in Proteobacteria after pneumococcal inoculation reflects an inflammation-driven anaerobic nitrate rich environment that favours these organisms over the Firmicutes that are limited to fermentation to derive their energy. The notable expansion of the OTU1 identified as an *Ochrobactrum* species that shows a strong negative correlation with the introduced SRL1 pneumococcal strain may also be because of this environmental change. *Ochrobactrum* species have been shown to have a very high ability to utilize nitrate, nitrite and other nitrogen containing compounds as electron acceptors [37]. A previous study of pneumococcal colonization found a drop in diversity after pneumococcal inoculation but not the other differences outlined above, probably because it utilised a 10 x greater inoculating dose of pneumococci [38].

How then does the pneumococcus establish itself in the nasal bacterial community and outcompete the other constituents, as seen in many of the inoculated animals studied here? That this was much more marked in the absence of IL-17 signalling implies IL-17 driven defence mechanisms are important in normal control of such colonization. Paradoxically, the lack of IL-17 signalling leads to expansion of Proteobacteria in the nose, which as outlined above would appear to have a significant energetic advantage over non-respiring pneumococci. One possibility is that pneumococci occupy a different anatomical niche from resident Proteobacteria. This then allows the Pneumococcus to proliferate free from competition from other members of the nasal microflora, and establish itself as the dominant bacterium within the nasal space. Whatever the location occupied by invading pneumococci, it is normally protected by IL-17 driven mechanisms, such as neutrophil influx and/or antimicrobial peptides, as colonization of pneumococci is much higher in the *Il17ra* KO mice.

The sparse correlation analysis of bacterial components before and after pneumococcal inoculation show some unexpected findings. Although strong co-operation between species has been thought to enhance community stability, both theoretical and experimental analysis have shown that weak interactions are likely to prevail in stable communities [39–41]. This is what we have observed in the resting nasal microbiome of both wild type and *Il17ra* KO animals. However, after pneumococcal inoculation we observed strong positive interactions within members of the Firmicutes and Proteobacteria groups, but strong antagonism between members of these groups. Promotion of other Firmicutes by pneumococcal colonization was unexpected, as bacteriocin production by the pneumococcus is thought to kill closely related Firmicutes and promote its own growth [22]. We have performed whole genome RNA sequencing of SRL1 within lung and pleura and have seen strong expression of the bacteriocin gene locus (data not shown). The mechanism underlying the positive co-correlation of Firmicutes with each other following pneumococcal colonization is thus not clear. One possibility is the production of carbohydrate energy sources from the degradation of mucin by pneumococci, which could then cross-feed other Firmicutes [42]. However, it calls into question the role of bacteriocins in the ability of pneumococci to colonize the nasopharynx. The mechanism underlying the positive co-correlations of members of the proteobacteria are also unclear, as this group too produces bacteriocins. The negative interactions between the Firmicutes and Proteobacteria we propose are due to the ability of Proteobacteria to respire using nitrate as outlined above. In both groups of mice, the alterations in interactions between the members of the microbiome were still evident 14 days after pneumococcal inoculation.

The lung microbiome of wild type animals showed no disruption following pneumococcal nasal colonization, but in the absence of IL-17 signalling, there was a significant alteration in the composition of the microflora. In those animals that had high levels of pneumococcal nasal colonization, there was a corresponding increase in the presence of the colonizing pneumococcus in the lung microbiome. Despite this, these animals did not show signs of clinical illness. Even in the absence of IL-17 signalling, therefore, there is an immune response within the lung that is sufficient to prevent the development of clinical disease despite the presence of the pathogenic pneumococcus.

In conclusion, we have shown here that IL-17 signalling influences the composition of the nasal but not the lung microbiome. Following pneumococcal colonization, there were marked changes in nasal microbiome composition, with wild type animals becoming to resemble the *Il17ra KO* animals, with an increase in Proteobacteria. In the lung, only the *Il17ra* animals showed altered microbiome composition, with the invading pneumococcus forming a considerable proportion of the recovered bacterial population in some animals despite absence of clinical disease. These results demonstrate the important effects of host cell IL-17 immunity not just on resisting an invading pathogen but also in altering the resident microbiome. Such changes may influence the ability of the invading pneumococcus to establish colonization, and potentially could be manipulated as a therapeutic intervention.

## Conclusions

We show here that the signalling from the IL-17 family cytokines profoundly influences the nasopharyngeal microbiome of experimental mice. Following colonization with the pathogen, *Streptococcus pneumoniae,* there is significantly enhanced recovery of this microbe from the nasopharynx of *Il17ra* knockout mice, and multiple changes in microbiome composition, even in animal without established pneumococcal colonization. Following pneumococcal inoculation, there are significant changes in interactions between different microbial groups, with positive interactions within members of the Firmicutes and Proteobacteria between themselves, and strong antagonism between these two groups. These results demonstrate that IL-17 plays a role not just in altering innate immune defence directly but also in nasopharyngeal microbiome composition that impacts upon the ability of the invading pneumococcus to establish colonization. These microbial interactions could potentially be manipulated to reduce pneumococcal colonization and disease.

## Methods

### Bacterial strains

The clinical strain SRL1 (serotype 3, ST180) was obtained from the Scottish Haemophilus, Legionella, Meningococcus and Pneumococcus Reference Laboratory.

### Animals

All mice were used between 8 and 12 weeks of age. *Il17ra* KO mice on a C57Bl/6 background [30] were from T. Mitchell, University of Glasgow and originally supplied by Jay Kolls; C57BL/6 mice bred in-house were used as wild-type controls. For the microbiome studies, wild type and Il17RAKO animals were cohoused for 6 weeks prior to the nasal sampling and pneumococcal colonization. Animal work was carried out under a Project Licence as required by UK Home Office regulations as well as scrutiny and approval by an Institutional Review board.

### Ethics

All animal work was carried out with the authorization of the UK Government Home Office, as approved by the awarding of a Project Licence Number 60/4361 and was additionally approved by the University of Glasgow Animal Use Ethics Review Board, with the same reference number. This legislation ensures that all animal work carried out is in accordance with the UK Law as set out in the Animals (Scientific Procedures) Act 1986 (ASPA). This Act adopts the International European Union regulations set out in European Directive 2010/63/EU on the protection of animals used for scientific purposes, to which all our animal work adhered.

### Bacterial Colonisation

Bacterial colonisation was carried out by gently restraining mice and placing 10μl of PBS containing 5 x 10^5^ cfu of SRL1 on the external nares so that it was aspirated into the nasal tract. Animals were not sedated during this procedure to minimise aspiration into the lower respiratory tract. Animals were monitored closely during experiments with monitoring of bodyweight and assessment for signs of illness.

### Bacterial Infection and quantification of IL-17 targets

To analyse the gene responses in wild type and IL-17RA knockout animals, we increased the dose of added bacteria by inoculating 10 μl of PBS containing 106 SRL1. 12 hours following inoculation, lungs were placed in RNA later solution (Life Technologies) and frozen at -80°C until use. After gentle thawing, lungs were transferred into a 2ml centrifuge tube with a 5mm steel bearing (Qiagen) and 1ml Trizol (Life Technologies). Samples were loaded into a TissueLyser LT (Qiagen) and pulsed at 50 Hz for 5 minutes. RNA was then extracted from the lung homogenate using a PureLink RNA Mini Kit (Life Technologies) and first strand cDNA generated using SuperScript First-Strand Synthesis System (Life Technologies) using oligoDT primers according to the manufacturer’s instructions.

PCR was then conducted using Fast SYBR Master Mix (Life Technologies) according to the manufacturer’s instructions using the following primers: TBP forward CCCCACAACTCTTCCATTCT, reverse GCAGGAGTGATAGGGGTCAT; β-defensin 4 forward TCTCTTCACATTTCTCCTGGTG, reverse TGGATTGTTGATAATTTGGGTAAA; S100A8 forward TGCGATGGTGATAAAAGTGG, reverse GGCCAGAAGCTCTGCTACTC; MIP-1α forward CGTTCCTCAACCCCCATC, reverse TGTCAGTTCATGACTTTGTCATCAT. Gene expression was compared to that of the housekeeping gene TATA-binding protein (TBP) and expressed as relative gene expression according to the ΔΔCT method.

### Respiratory tract sampling and culture

Mice were culled by inhalation of CO_2_. The external nares were carefully cleaned and allowed to dry. The trachea was exposed and incised to allow the placement of a fine bore pastette. To wash the nasal passages, the pastette tip was advanced via the trachea into the posterior nasopharynx until resistance was felt and 1 ml of phosphate buffered saline (PBS) expressed through the nose. Broncho-alveolar lavage was performed with 1ml of PBS. Pneumococci were enumerated by serial dilution onto 5% horse blood agar and incubated overnight in 5% CO_2_. For estimation of nasal neutrophil content, the nasal washes were washed three by centrifugation at 400g for 5 minutes in PBS before resuspending in FACS buffer (BD Biosciences). Cells were stained with anti-Gr-1 antibody (clone RB6-8C5, Biolegend) conjugated to PE diluted 1:1000 with FACS buffer for 30 minutes at 37°C and then analysed using a CyAN ADP Flow cytometer (Becton Dickinson).

### 16S DNA amplification and sequencing

Genomic DNA was extracted from nasal washes and BALF using a Qiagen DNeasy Blood and Tissue Mini Kit according to the manufacturer’s instructions using the suggested modification to enhance lysis of gram-positive organisms and eluted into 100 μl. After extraction, the V1-V2 region of the 16S RNA was amplified using NEB Next High Fidelity 2x PCR Master Mix (New England Biolabs) using 10 μl of genomic DNA and 12.5 pmol of the high performance liquid chromatography barcoded primers shown in Additional file 2: Table S1 (Eurofins Genomics). Thus, PCR products from the different animals and time points could be identified after sequencing by their associated barcode, allowing pooling of samples for sequencing. PCR was conducted using an initial denaturation period of 30 seconds at 98 °C followed by 25 cycles consisting of denaturation for 10 seconds at 98 °C, annealing for 30 seconds at 58 °C, and extension for 30 seconds at 72 °C before a final extension for 1 minute at 72 °C.

Presence of a single PCR product at the expected band size of 440 bp was confirmed by 1.8% agarose gel electrophoresis before purification of the PCR product using a Qiagen PCR purification kit according to the manufacturer’s instructions. Samples from different animals and time points were then pooled.

Sequencing was performed by the Centre for Genomic Research (CGR), University of Liverpool. Paired end (2x250bp) sequencing of pooled amplicon sample on the Illumina MiSeq platform was conducted using higher levels of Phix to increase diversity. Sequences were demultiplexed by the CGR.

Sequences were processed using a processing pipeline. Briefly, sequences were trimmed using Sickle [43] and forward and reverse reads were overlapped using PANDASEQ [44] with a minimum overlap of 50bp to assemble the forward and reverse reads into a single sequence spanning the entire V1-V2 region [45]. Sequences were then dereplicated and chimeric sequences removed using UCHIME [46]. Operational taxonomic units (OTUs) were then generated using UPARSE [47] and taxonomic assignments were generated using the standalone RDPclassifier v2.6 [48, 49] with the default --minWords option of 5. Tax4Fun [50] was used to predict the functional capabilities of microbial communities based on 16S rRNA datasets after blasting the OTUs against the Silva database.

### *Detection of pneumococci using PCR amplification of* cpsA

DNA was extracted from pneumococci using a DNeasy Blood and Tissue Kit (Qiagen) according to the manufacturer’s instructions using the protocol for gram positive bacteria. PCR was carried out using Qiagen Multiplex PCR mastermix according to the manufacturer’s instructions. Primers for the cpsA gene were: GCAGTACAGCAGTTTGTTGGACTGACC (forward) and GAATATTTTCATTATCAGTCCCAGTC (reverse). After an initial incubation at 95°C for 15 minutes to activate the DNA polymerase, 35 cycles were performed consisting of denaturation at 95°C for 30 seconds, annealing at 54°C for 90 seconds and extension at 72°C for 60 seconds. A terminal extension step of 10 minutes was included. Final mixtures were run on a 2% agarose gel at 100V for 2 hours. The amplification with these primers generates a 160 bp band.

### Statistics

Statistical analysis was conducted using R [51]. Nonmetric Distance Scaling (NMDS) plots of community data (OTUs at 3% divergence) using Bray-Curtis dissimilarity between samples were produced with the metamds function in the Vegan package. Samples were grouped for different conditions and the mean ordination value and spread of points shown using Vegan’s ordiellipse function. Differences between groups were evaluated using PERMANOVA in the adonis function in the Vegan package [25]. Sparse correlation analysis was performed using Spar-CC [52] as implemented in R [53]. Significance testing of correlation co-efficients was calculated by re-sampling (with replacement) the normalised count data to produce 100 resampled count matrices for which new Spar-CC correlations were calculated. Pseudo *p* values were than calculated as the proportion of these resampled Spar-CC correlation co-efficients that were more extreme than the original value; *p* < 0.05 was taken as significant.

## Additional files

Additional file 1: Figures S1-4. Additional figures S1- S5. (PDF 1437 kb)

Additional file 2: Table S1. Additional Table S1. (CSV 48 kb)

Additional file 3: Raw OTU counts from wild type animals at baseline. (CSV 220 kb)

Additional file 4: Raw OTU counts from wild type animals at 3 days after colonization. (DOCX 135 kb)

Additional file 5: Raw OTU counts from wild type animals at 7days after colonization. (CSV 66 kb)

Additional file 6: Raw OTU counts from wild type animals at14 days after colonization. (CSV 48 kb)

Additional file 7: Raw OTU counts from *Il17ra* animals at baseline. (CSV 48 kb)

Additional file 8: Raw OTU counts from *Il17ra* animals at 3 days after colonization. (CSV 49 kb)

Additional file 9: Raw OTU counts from *Il17ra* animals at 7 days after colonization. (CSV 70 kb)

Additional file 10: Raw OTU counts from *Il17ra* animals at 14 days after colonization. (CSV 49 kb)

Additional file 11: Taxonomic allocation of OTUs. (CSV 48 kb)

## Abbreviations

IL: interleukin
NMDS: non-metric multidimensional scaling
OTU: operational taxonomic unit
