# Supplementary material for: IL-17 signalling restructures the nasal microbiome and drives dynamic changes following Streptococcus pneumoniae colonization
Source: BMC Genomics. 2017 Oct 23;18:807. doi: 10.1186/s12864-017-4215-3 (PMC5651609; doi:10.1186/s12864-017-4215-3)

Supporting Information

Figure S1. **Assessment of Sequencing Depth**

A. Rarefaction curves for upper and lower respiratory tract samples by time of colonisation. Time in days represents time after colonisation. B. Percentage of OTUs detected per sample using the abundance-based coverage estimator (ACE). (black: wild type, red: *Il17ra* KO).

A

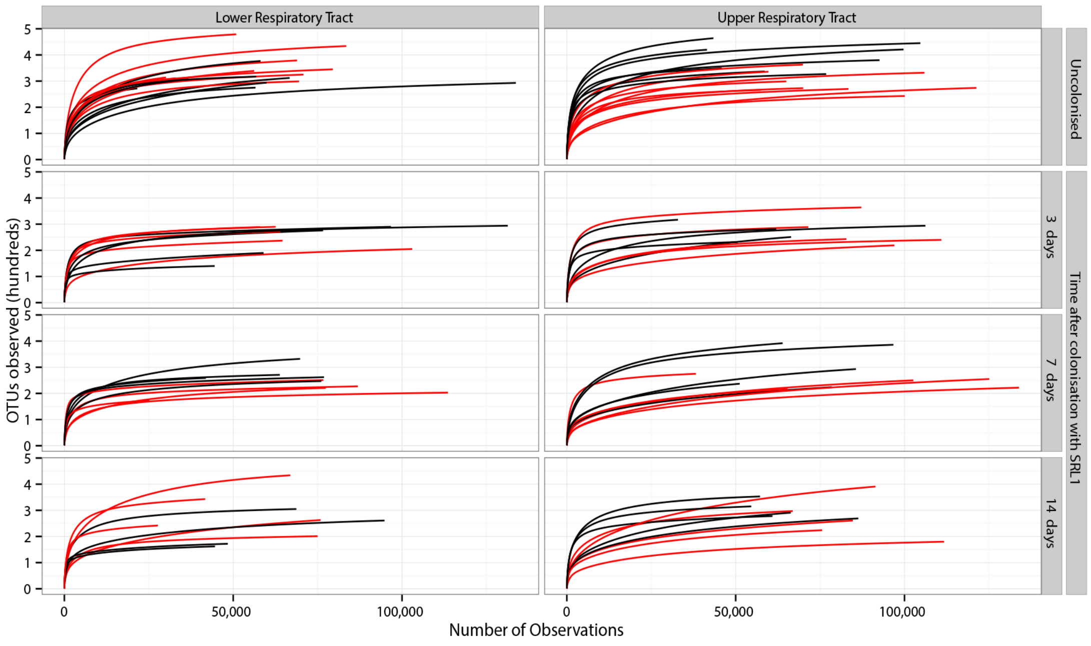

B

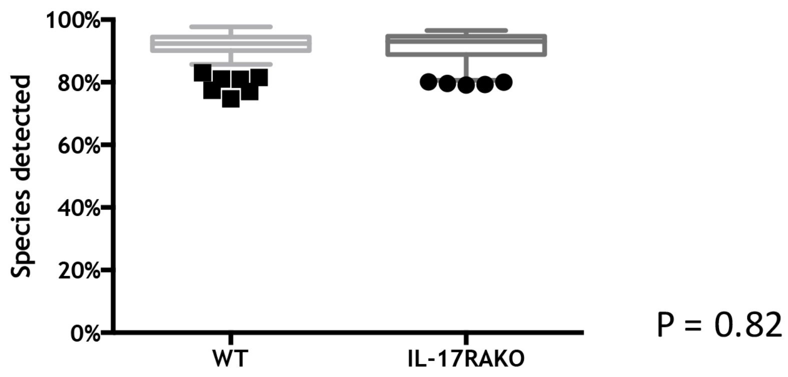

Figure S2. **Gene expression of IL-17 target genes in SRL1 infection.**

Gene expression was quantified by RT-PCR in wild type and IL17RA$^{-/-}$ animals 12 hours after infection with 10^6 cfu SRL1. Targets are Beta-defensin 4 (BetaDef4), S100A8, and macrophage inhibitory protein 1 alpha (MIP-1alpha). Gene expression is normalised to expression of TATA-binding protein (TBP) and expressed as a ratio of expression in uninfected wild-type animals (n=3) ± SEM. P values calculated by Student's t-test : * <0.05, ** <0.01, *** <0.001.

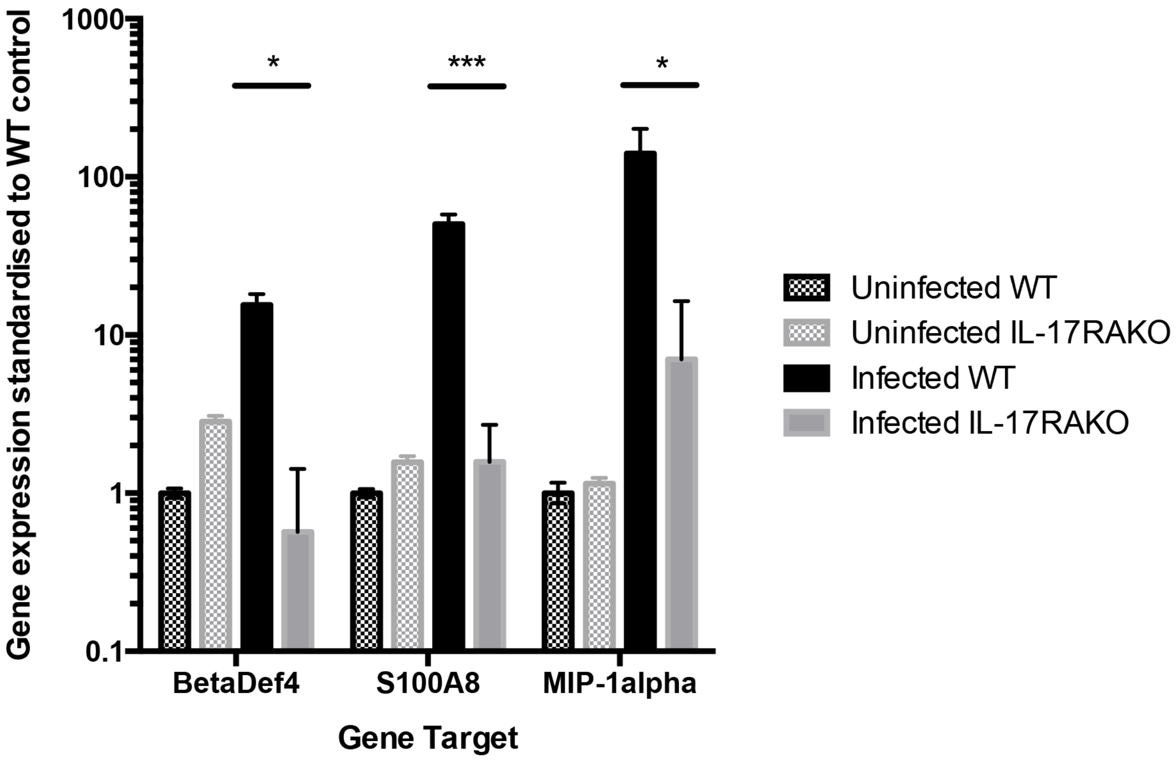

Figure S3. **Neutrophils within nasal wash**

Each symbol shows the value from an individual animal; line indicates median. Values from infected animals were taken 7 days after inoculation. Difference between wild type (WT) and *Il17ra* KO animals significant (Mann Whitney test).

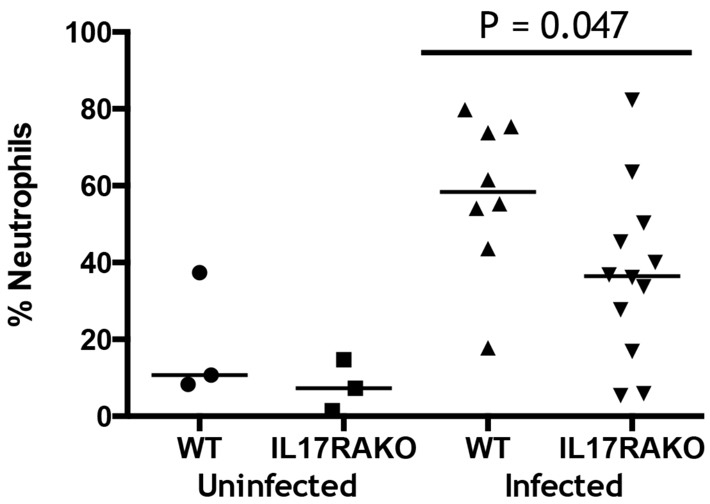

Figure S4. **Presence of *S. pneumoniae* in nasal washes**

Representative PCR of the pneumococcal *cps* gene on nasal lysates. + denotes positive control reactions. M is size ladder.

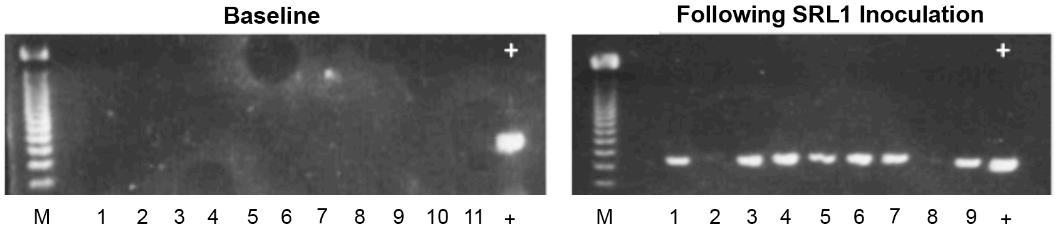

Figure S5. **Changes in bacterial composition of nasal microbiome following pneumococcal colonization**

(A). Proportion of Proteobacteria within microbiome at various times after pneumococcal inoculation. Points are means of 5-9 observations, error bars are SEM. ** significantly different form the value at time 0 ($p < 0.01$, 2 sample t test).

(B). Abundance of Firmicutes within the nasal microbiome at various levels of pneumococcal abundance. Each point is from a single animal. The dotted line shows the median value for all animals for each OTU. The high, medium and low abundance classifications of OTU2 correspond to the top, middle and bottom tertiles of OTU2 abundance across all time points. Colour code for different family members is shown. The correlation between abundance of each OTU and level of pneumococcal colonization was significant ($p<0.0001$).

A

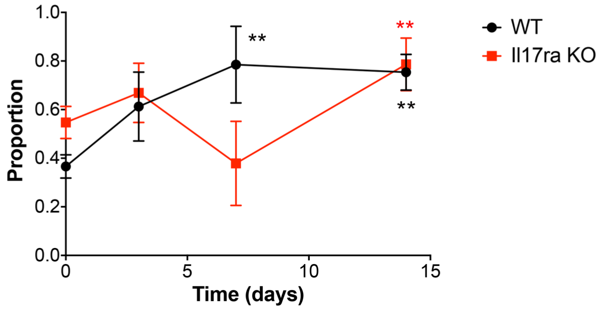

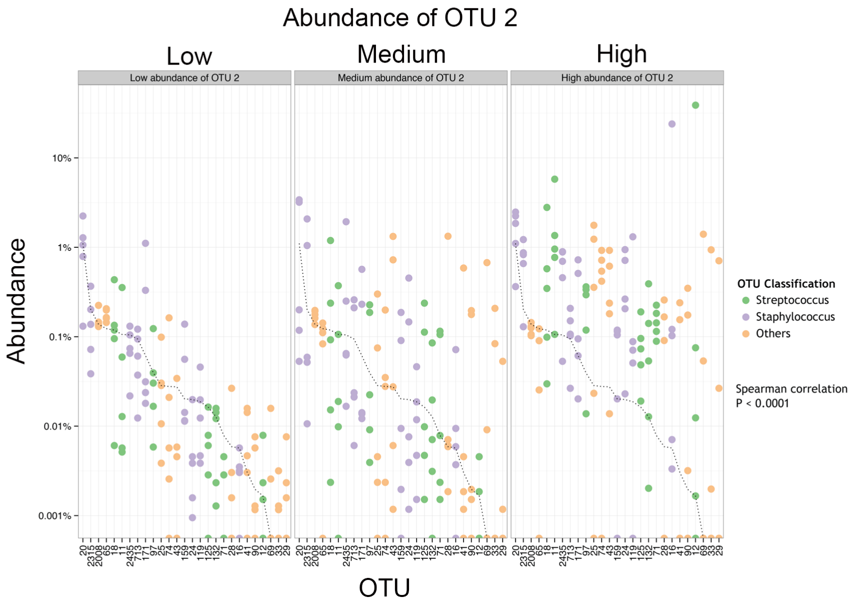

Supplement: Supplementary file 1 — Additional figures S1- S5. (PDF 1437 kb) [file 12864_2017_4215_MOESM1_ESM.pdf]
