# Supplementary material for: IL-17 signalling restructures the nasal microbiome and drives dynamic changes following Streptococcus pneumoniae colonization
Source: BMC Genomics. 2017 Oct 23;18:807. doi: 10.1186/s12864-017-4215-3 (PMC5651609; doi:10.1186/s12864-017-4215-3)
Supplement: Supplementary file 4 — Raw OTU counts from wild type animals at 3 days after colonization. (DOCX 135 kb) [file 12864_2017_4215_MOESM4_ESM.docx]

Table S1. **Primers used for PCR.**

Top two rows contain generic primer sequence. Reverse primer contains one of the barcodes shown in the remaining rows below.

| Forward Primer | AATGATACGGCGACCACCGAGATCTACAC TATGGTAATT CC AGMGTTYGATYMTGGCTCAG | | | | |
| --- | --- | --- | --- | --- | --- |
| Reverse Primer | CAAGCAGAAGACGGCATACGAGAT XXXXXXXXXXXX AGTCAGTCAG AA GCTGCCTCCCGTAGGAGT | | | | |
| Primer | Barcode | Primer | Barcode | Primer | Barcode |
| 338rcbc1 | ACGAGACTGATT | 338rcbc33 | CAACTCCCGTGA | 338rcbc65 | ACTTCCAACTTC |
| 338rcbc2 | GCTGTACGGATT | 338rcbc34 | TTGCGTTAGCAG | 338rcbc66 | CTCACCTAGGAA |
| 338rcbc3 | ATCACCAGGTGT | 338rcbc35 | TACGAGCCCTAA | 338rcbc67 | GTGTTGTCGTGC |
| 338rcbc4 | TGGTCAACGATA | 338rcbc36 | CACTACGCTAGA | 338rcbc68 | CCACAGATCGAT |
| 338rcbc5 | ATCGCACAGTAA | 338rcbc37 | TGCAGTCCTCGA | 338rcbc69 | TATCGACACAAG |
| 338rcbc6 | GTCGTGTAGCCT | 338rcbc38 | ACCATAGCTCCG | 338rcbc70 | GATTCCGGCTCA |
| 338rcbc7 | AGCGGAGGTTAG | 338rcbc39 | TCGACATCTCTT | 338rcbc71 | CGTAATTGCCGC |
| 338rcbc8 | ATCCTTTGGTTC | 338rcbc40 | GAACACTTTGGA | 338rcbc72 | GGTGACTAGTTC |
| 338rcbc9 | TACAGCGCATAC | 338rcbc41 | GAGCCATCTGTA | 338rcbc73 | ATGGGTTCCGTC |
| 338rcbc10 | ACCGGTATGTAC | 338rcbc42 | TTGGGTACACGT | 338rcbc74 | TAGGCATGCTTG |
| 338rcbc11 | AATTGTGTCGGA | 338rcbc43 | AAGGCGCTCCTT | 338rcbc75 | AACTAGTTCAGG |
| 338rcbc12 | TGCATACACTGG | 338rcbc44 | TAATACGGATCG | 338rcbc76 | ATTCTGCCGAAG |
| 338rcbc13 | AGTCGAACGAGG | 338rcbc45 | TCGGAATTAGAC | 338rcbc77 | AGCATGTCCCGT |
| 338rcbc14 | ACCAGTGACTCA | 338rcbc46 | TGTGAATTCGGA | 338rcbc78 | GTACGATATGAC |
| 338rcbc15 | GAATACCAAGTC | 338rcbc47 | CATTCGTGGCGT | 338rcbc79 | GTGGTGGTTTCC |
| 338rcbc16 | GTAGATCGTGTA | 338rcbc48 | TACTACGTGGCC | 338rcbc80 | TAGTATGCGCAA |
| 338rcbc17 | TAACGTGTGTGC | 338rcbc49 | GGCCAGTTCCTA | 338rcbc81 | TGCGCTGAATGT |
| 338rcbc18 | CATTATGGCGTG | 338rcbc50 | GATGTTCGCTAG | 338rcbc82 | ATGGCTGTCAGT |
| 338rcbc19 | CCAATACGCCTG | 338rcbc51 | CTATCTCCTGTC | 338rcbc83 | GTTCTCTTCTCG |
| 338rcbc20 | GATCTGCGATCC | 338rcbc52 | ACTCACAGGAAT | 338rcbc84 | CGTAAGATGCCT |
| 338rcbc21 | CAGCTCATCAGC | 338rcbc53 | ATGATGAGCCTC | 338rcbc85 | GCGTTCTAGCTG |
| 338rcbc22 | CAAACAACAGCT | 338rcbc54 | GTCGACAGAGGA | 338rcbc86 | GTTGTTCTGGGA |
| 338rcbc23 | GCAACACCATCC | 338rcbc55 | TGTCGCAAATAG | 338rcbc87 | GGACTTCCAGCT |
| 338rcbc24 | GCGATATATCGC | 338rcbc56 | CATCCCTCTACT | 338rcbc88 | CTCACAACCGTG |
| 338rcbc25 | CGAGCAATCCTA | 338rcbc57 | TATACCGCTGCG | 338rcbc89 | CTGCTATTCCTC |
| 338rcbc26 | AGTCGTGCACAT | 338rcbc58 | AGTTGAGGCATT | 338rcbc90 | ATGTCACCGCTG |
| 338rcbc27 | GTATCTGCGCGT | 338rcbc59 | ACAATAGACACC | 338rcbc91 | TGTAACGCCGAT |
| 338rcbc28 | CGAGGGAAAGTC | 338rcbc60 | CGGTCAATTGAC | 338rcbc92 | AGCAGAACATCT |
| 338rcbc29 | CAAATTCGGGAT | 338rcbc61 | GTGGAGTCTCAT | 338rcbc93 | TGGAGTAGGTGG |
| 338rcbc30 | AGATTGACCAAC | 338rcbc62 | GCTCGAAGATTC | 338rcbc94 | TTGGCTCTATTC |
| 338rcbc31 | AGTTACGAGCTA | 338rcbc63 | AGGCTTACGTGT | 338rcbc95 | GATCCCACGTAC |
| 338rcbc32 | GCATATGCACTG | 338rcbc64 | TCTCTACCACTC | 338rcbc96 | TACCGCTTCTTC |
